# Annual-resolution evidence for aridity spikes at the onset of the Last Glacial Inception in an Eifel maar lake sediment

**DOI:** 10.1038/s41598-026-64586-6

**Published:** 2026-08-15

**Authors:** Frank Sirocko, Frederik Krebsbach, Christian Forster, Frank Dreher, Martin Grosjean, Lucas Silva, Dana F. C. Riechelmann, Klemens Seelos, Ralf Schiebel

**Affiliations:** 1 Institute of Geosciences, Johannes Gutenberg-University Mainz, Mainz, Germany; 2 University of Bern, Oeschger Centre for Climate Change Research & Institute of Geography, Bern, Switzerland; 3 Climate and Environmental Physics, University of Bern, Bern, Switzerland; 4 Max Planck Institute for Chemistry, Mainz, Germany

**Keywords:** Climate sciences, Ecology, Ecology, Environmental sciences, Natural hazards

## Abstract

This study presents an annually laminated sediment record from the infilled maar lake at Hoher List in the Eifel volcanic field (Germany), documenting the “Last Glacial Inception” (LGI) at the end of the Eemian interglacial. The landscape of the late Eemian was dominated by a mixed forest containing *Abies alba* (silver fir). The collapse of this forest occurred within only a few years and was accompanied by frequent wildfires, aeolian dust deposition, a decline of all thermophilous tree species, and the disappearance of *Abies*, all indicating increasing aridity. The transition unfolded in three distinct phases. LEAP-I (Late Eemian Aridity Pulse I) lasted approximately 250 years and was followed by a 750-year recovery phase during which the interglacial forest returned. LEAP-II was characterized by comparable arid conditions but persisted for at least 750 years, followed by a 350-year phase of boreal forest development. The final transition to stadial conditions occurred during LEAP-III, marked by a pronounced lake-level decline, expansion of grassland, scattered birch and pine, and reduced summer temperatures, likely corresponding to North Atlantic cold event C25. The LGI lasted at least 2700 years, whereas individual aridification pulses developed within decades, indicating rapid aridification events superimposed on a long-term cooling trend.

**Supplementary Information:**

The online version contains supplementary material available at 10.1038/s41598-026-64586-6.

## Introduction

The transition from the Last Interglacial (Marine Isotope Stage 5e; Eemian) into the subsequent cold phase represents a key interval for understanding how interglacial climates terminate and evolve towards glacial inception. Syntheses of high-resolution terrestrial and marine archives constrain the duration of the Last Interglacial to approximately 129,000–116,000 BP and document a staged pattern of environmental deterioration^1^. The synthesis by Tzedakis et al.^2^ established a robust chronological framework linking ocean, cryosphere, and vegetation records and demonstrated that the demise of the interglacial was neither gradual nor spatially uniform.

Marine records from the North Atlantic highlight the importance of oceanic feedbacks during this interval. A weakening of the Atlantic Meridional Overturning Circulation (AMOC) near 118,000 BP coincided with cooling of sea-surface waters and the onset of enhanced iceberg discharge^3,4^. Instabilities in North Atlantic Deep Water formation pre-dated full glacial inception, indicating that the ocean acted as a key amplifier rather than a passive responder^5^. On land, multiple archives across central and northern Europe indicate abrupt aridification and ecological deterioration. Alpine speleothems record a decline in δ¹⁸O values, reflecting reduced temperatures and precipitation across central Europe^6^. In western Europe, growth of the Han-9 stalagmite from Belgium ceased at 117,300 BP (+ 700/−1000 years)^7,8^.

A comparison of different paleorecords for this time reveal seasonally decoupled temperature trends, with winter cooling preceding summer temperature declines^9^. Differences between the local weather in northern, central, and southern Europe are to be expected if the forcing of the abrupt transitions was associated with the North Atlantic hydrography, analogous to modern North Atlantic Oscillations (NAO), which cause opposing weather conditions between northern and southern Europe^10^.

Cryospheric and atmospheric records provide complementary evidence for this transition, commonly referred to as the Last Glacial Inception (LGI). The NEEM ice core from Greenland documents a rapid reorganization of dust provenance and atmospheric circulation between 119,000 and 117,000 BP^11,12^.

Collectively, these records depict a tightly coupled climate system in which declining Northern Hemisphere summer insolation initiated feedbacks among the ocean, atmosphere, and cryosphere^13^. A weakening of the AMOC and the expansion of sea ice likely reduced northward heat and moisture transport, thereby reinforcing continental aridity^3^.

**Fig. 1:**
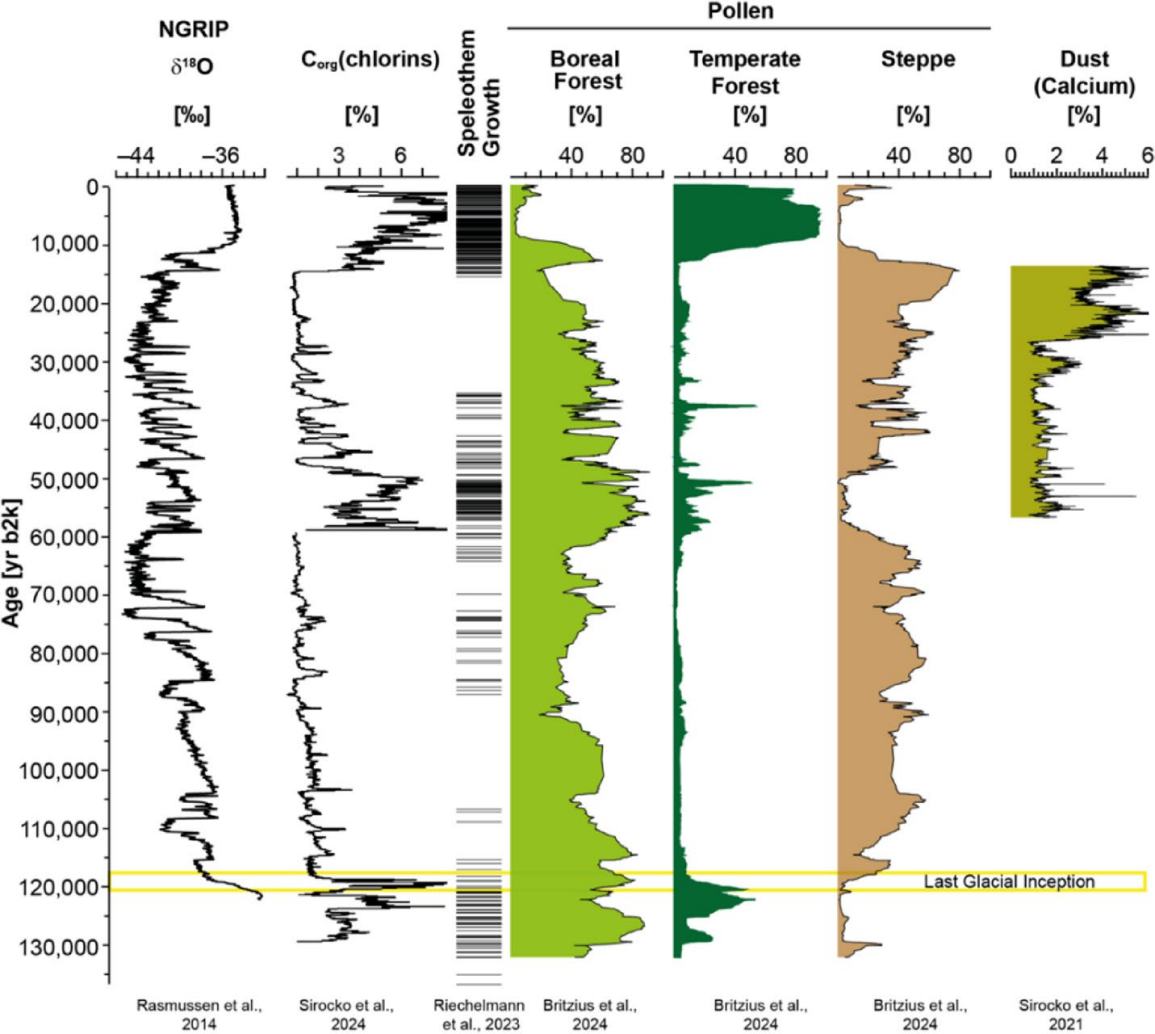
Overview of ELSA sediment cores, age models, and regional climate archives used for reconstruction of environmental and climatic change during the last glacial cycle. The synthesis integrates published ELSA records of climate, vegetation, and volcanic activity from the Eifel region covering the past 130,000 years^15,17,19,22^ and compares them with NGRIP δ¹⁸O records^21^ and speleothem growth phases from central Europe^22^.

A laminated sediment record (core HL2) from the infilled maar lake at Hoher List was the first archive to reveal the decadal-scale structure of the LGI. This floating annual-resolution varve record documented an interval of pronounced aridification that was termed the Late Eemian Aridity Pulse (LEAP)^14^. To further investigate the annual-scale structure of this event, a new sediment core (HL4) was recovered within the framework of the Eifel Laminated Sediment Archive (ELSA) project in 2012. HL4 forms part of a regional stack of maar sediment cores used to reconstruct environmental conditions, vegetation dynamics, and volcanic activity in the Eifel region over the past 130,000 years^15–20^ (Fig. 1); the chronology is tuned to the NGRIP ice core^21^.

The lithology and Eemian pollen succession at Hoher List are highly similar to records from the infilled maar lakes of Jungferweiher and Eigelbach (Supplementary Fig. 1). All three sites document the transition from the Eemian into the subsequent colder phases associated with the LGI and display comparable lithological characteristics throughout this interval (Supplementary Figs. 2–5). The Hoher List record is therefore considered representative of the wider Eifel region, which closely reflects climatic developments across central Europe^23^.

**Fig. 2:**
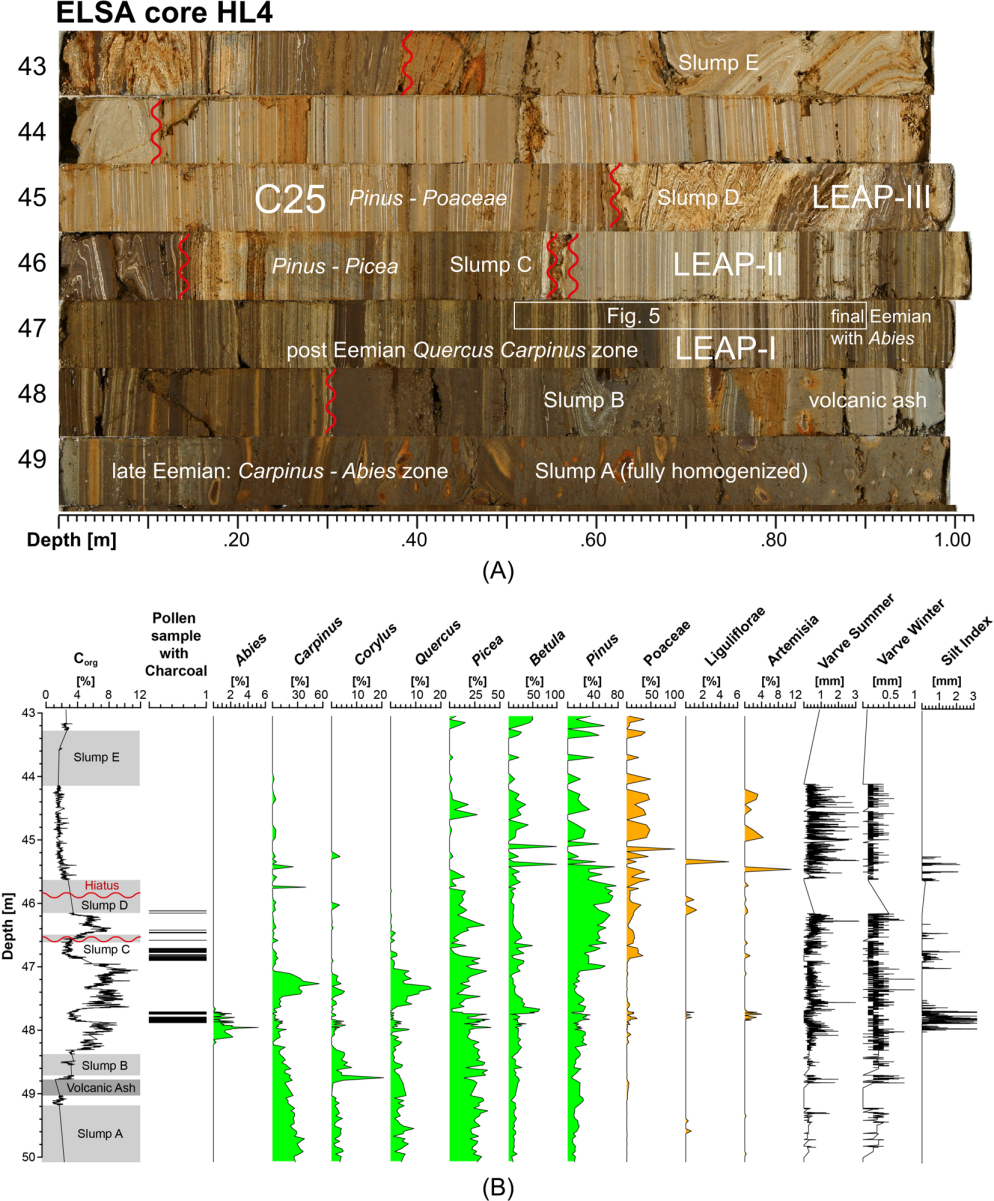
A. Photograph of the HL4 sediment sequence spanning the transition from the Eemian Interglacial to the subsequent phase of the Last Glacial Inception. Stratigraphic units, slumps, and environmental events discussed in the text are indicated. **B**. C_org_ (*chlorins*) and selected pollen records from core HL4 corresponding to the sediment interval shown in panel A.

Annual laminations (varves) are preserved in sediments from the infilled maar lakes at Hoher List and Eigelbach^14^. However, only the new HL4 core allows continuous varve counting across the LGI interval (Fig. 2). HL4 was recovered from the central part of the infilled maar basin, where sediment deposition occurred under seasonally anoxic conditions similar to those observed in modern Eifel maar lakes^24^. The upper water column of modern maar lakes becomes thermally stratified during summer, providing favourable conditions for diatom productivity and the accumulation of organic carbon (C_org_ *chlorins*). Elevated summer temperatures also promote precipitation of microcalcite particles. Consequently, seasonal laminations and organic-matter preservation primarily depend on reduced dissolved oxygen concentrations in bottom waters during periods of summer stratification. A disadvantage of coring the basin centre is the increased susceptibility to slumping. While varve preservation is generally excellent at this location, slumps are also preferentially focused towards the deepest part of the basin. These mass-movement deposits originate from erosion of sediment along the lake margins during lake-level fluctuations and may result in both sediment loss and redeposition. Core HL4 records evidence of both processes but also provides substantially improved varve preservation compared with core HL2. The new HL4 record demonstrates that the Late Eemian Aridity Pulse consisted of three distinct events, here termed LEAP-I, LEAP-II and LEAP-III (Fig. 2).

It is the goal of this study to provide inside into the succession and duration of these events on an indeed paleoweather scale. Accordingly, the paper leaves the time scale of classical paleoclimate studies and starts to describe weather structures, which can be used to start a discussion on the cause of the LGI and its potential forcing.

## Materials and methods

### Drillings

Sediment cores were recovered using wire-line drilling technology. Core locations at Hoher List, Jungferweiher, and Eigelbach are shown in Supplementary Fig. 1, and the corresponding coordinates are listed in Table 1.

**Table 1:** Sites and core locations.

Infilled maar lakes	Core	Core depth [m]	Height above sea level [m]	Coordinates East (WGS84)	Coordinates North (WGS84)
Eigelbach	EI2	75.00	423.50	6.589884952	50.18582631
Hoher List	HL4	62.00	400.00	6.835054596	50.16435909
Jungferweiher	JW3	156.00	432.00	6.975028664	50.21973162

### Dating

**Fig. 3:**
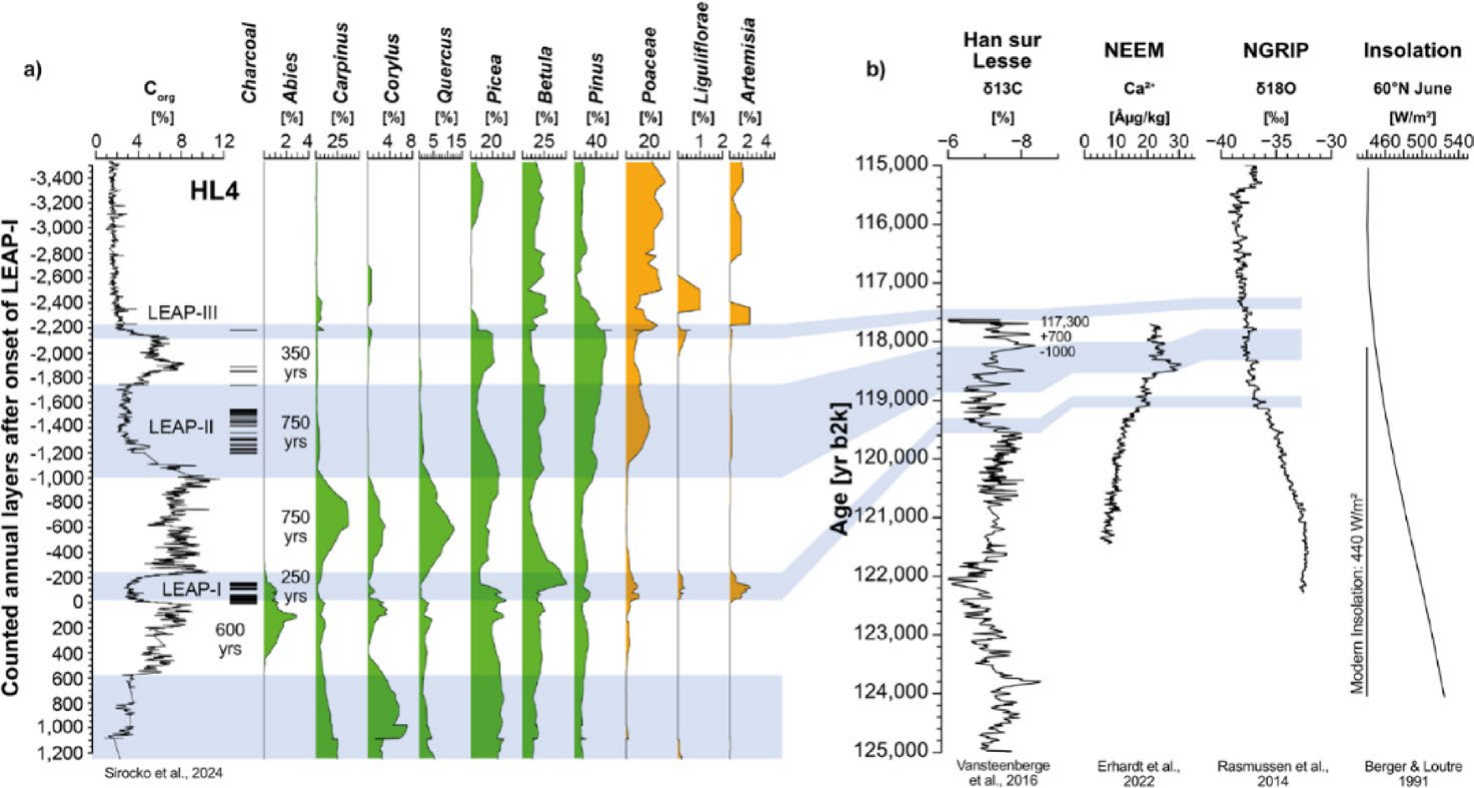
**(A)** Floating varve chronology of core HL4 showing C_org_ (*chlorins*), selected pollen taxa, charcoal, and the principal environmental phases of the terminal Eemian and the Last Glacial Inception. The onset of LEAP-I is defined as year zero of the floating chronology. **(B)** Comparison of the HL4 record with major climate archives spanning 112,000–125,000 yr b2k. Shown are June insolation at 60°N^27^, the HAN-9 stalagmite δ¹³C record from Belgium^7^, NGRIP δ¹⁸O^21^, and NEEM Ca concentrations^28^ as a proxy for atmospheric dust variability.

The first age estimates for cores HL2 and JW2 were presented by Sirocko et al.^14^. Infrared stimulated luminescence (IRSL) dating of feldspars from sand-sized dust layers yielded ages between 88,000 and 117,000 BP, although associated uncertainties range from 10,000 to 20,000 years^25^. A tephra layer located close to the luminescence-dated interval exhibits the characteristic mineralogical and geochemical composition of the Dümpelmaar Tephra, which has been dated by the ⁴⁰Ar/³⁹Ar method to 116,000 ± 16,000 BP at its eruption site^26^. Although these chronological constraints are associated with considerable uncertainties, they support the assignment of the three interglacial pollen records shown in Supplementary Figs. 6–8 to the Last Interglacial (Eemian). Based on these stratigraphic constraints, the floating HL4 varve chronology required an external age anchor. Several potential tie points involving the onset of LEAP-I and LEAP-III were compared with abrupt changes observed in Greenland and Antarctic ice-core records; however, none of these correlations proved unequivocal. The ²³⁰Th/U-dated speleothem record from Han-sur-Lesse Cave in Belgium^8^ was therefore used to anchor the most pronounced environmental change associated with LEAP-III, namely the cessation of speleothem growth and the marked increase in stalagmite δ¹³C values (Fig. 3). Vansteenberge et al.^8^ dated the final cessation of stalagmite growth, interpreted as a stop in drip-water supply, to 117,300 + 700/−1000 years using the ²³⁰Th/U disequilibrium method. The pronounced aridity signal recorded in the speleothem is considered likely synchronous with the major lake-level decline associated with LEAP-III. This event therefore serves as the anchor point for the late Eemian HL4 varve chronology (Fig. 3).

### C_org_*(chlorins)*

Variations in C_org_ (*chlorins*) provide the first clear indication of changing environmental conditions in the late Eemian maar lake. In modern maar lakes, organic carbon is produced primarily by phytoplankton, mainly chrysophytes and diatoms, during summer stratification. Organic matter produced in the warm surface waters settles to the lake floor and is preserved in anoxic bottom waters as seasonal varves, a process well documented in modern lake systems^24^.

The concentration of C_org_ (*chlorins*) therefore reflects both the production and preservation of organic matter. Primary productivity depends mainly on surface-water temperature and nutrient availability, whereas degradation of sinking biomass requires oxygenation of bottom waters. Such oxygenation can occur when summer stratification is disrupted by cooling and convective overturn. Accordingly, low C_org_ (*chlorins*) concentrations may reflect repeated disruption of summer stratification caused by intense cold winds during late spring or by cool summer conditions. Both mechanisms would promote oxidation of settling organic matter and result in the light-coloured sediment layers that characterize all LEAP intervals.

### Lithology and Logging

All sediment cores display the characteristic light-coloured layers associated with the LEAP phases, which are clearly visible in the core photographs presented in Supplementary Figs. 2–5. All cores were analysed at 1-mm resolution using In Situ Reflectance Spectroscopy (ISRS)^29^.

Absorption at 670 nm is caused by chlorophyll a, chlorophyll b, and their degradation products, which are derived primarily from diatom productivity. The resulting sedimentary signal is expressed as C_org_ (*chlorins*); further methodological details are provided by Sirocko et al.^15^.

### Pollen

Pollen samples were prepared following established laboratory procedures^30,31^; additional methodological details for ELSA pollen samples are provided by Britzius et al.^19^. Stratigraphic interpretation of the pollen records follows the classical Eemian pollen succession described by Müller^32^, Menke^33^, Zagwijn^34^, Woillard^35^, and Litt et al.^36^. The oldest pollen zones are characterized by *Pinus* and *Corylus*, followed by assemblages dominated by *Corylus*, *Quercus*, *Ulmus*, and *Tilia*. These successions are clearly recognizable at all three study sites (Supplementary Figs. 6–8). The subsequent *Carpinus–Abies* zone is partly homogenized by the large, folded slump deposits A and B (Fig. 2). Nevertheless, the overall palynological succession identified for the Eemian is highly similar at Hoher List, Jungferweiher, and Eigelbach (Supplementary Figs. 6–8) for the interval preceding Slump A.

Pollen data for the Eemian sections of cores HL2, EI2, and JW3 were previously presented by Sirocko et al.^14,25,37^ and were subsequently incorporated into the ELSA pollen stack of Britzius et al.^19^. The pollen counts for the LGI interval of core HL4 are provided in the Supplementary Data accompanying this study.

### Varves

Annual laminations (varves) ranging from 0.2 to 2 mm in thickness are visible both in the core photographs (Supplementary Figs. 2–5) and in petrographic thin sections (Supplementary Figs. 9 and 10). Varves were counted on 10-cm-long petrographic thin sections using a binocular microscope at 20× magnification.

The annual layers consist mainly of diatom cysts, microcarbonates, and dark brown plant remains. Associated clay particles and humic substances dominate the winter component of the varves. Summer calcite layers, precipitated from warm surface waters, are readily identified under polarized light (Supplementary Fig. 10). Quartz grains ranging from silt to fine-sand size and black charcoal particles are characteristic components of the LEAP intervals. The thickness of seasonal sublayers, the abundance of microcalcite, and grain-size characteristics vary considerably from year to year. Consequently, only the two dominant varve types characteristic of the late Abies forest phase and LEAP-I are illustrated in Supplementary Fig. 10.

### Grain size

**Fig. 4:**
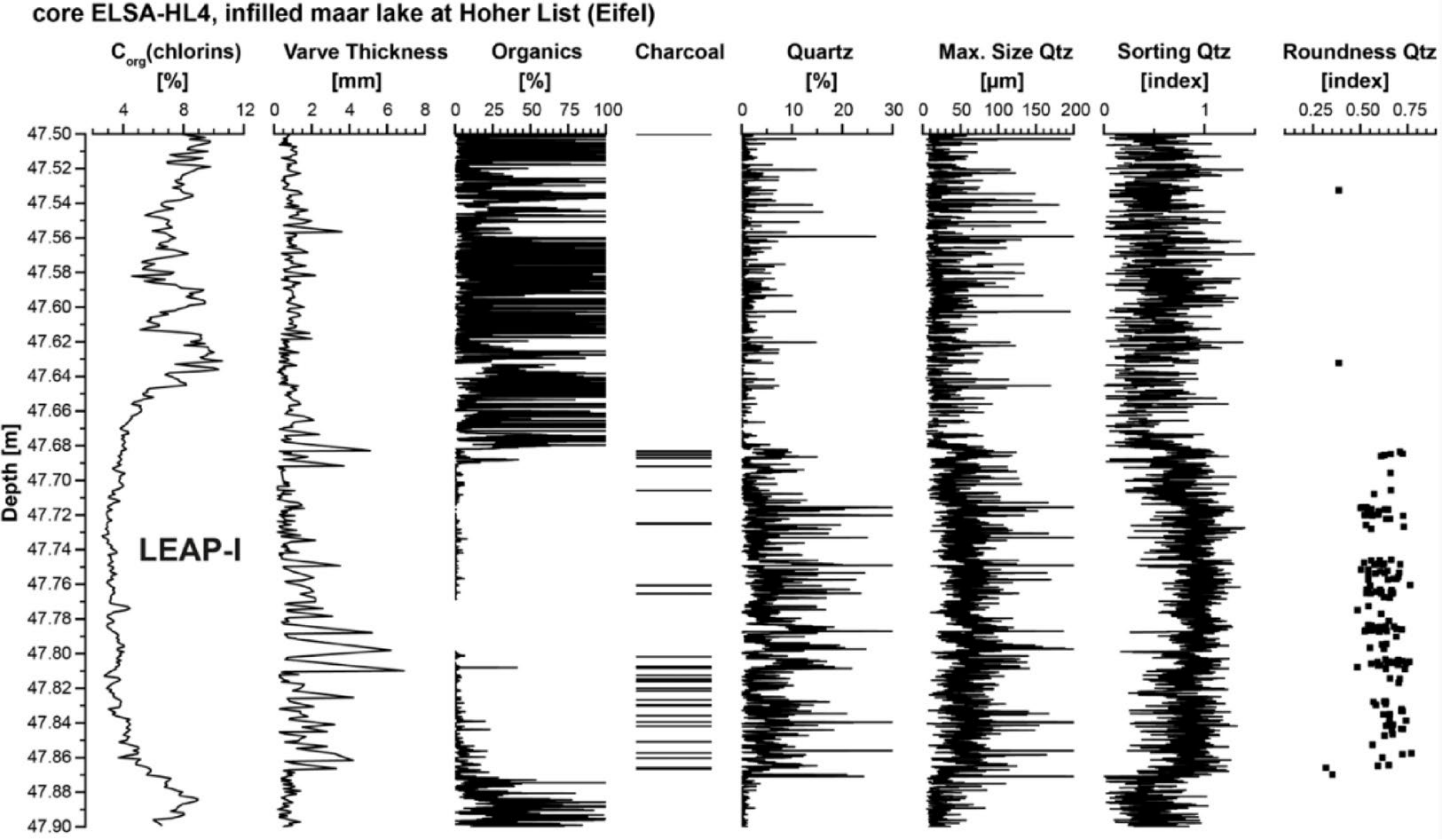
High-resolution proxy records from the LEAP-I interval of core HL4, including C_org_ (*chlorins*), varve thickness, charcoal abundance, organic particles, and quartz grain-size parameters derived from the RADIUS image-analysis method. The records illustrate the onset and development of arid conditions during LEAP-I.

**Fig. 5:**
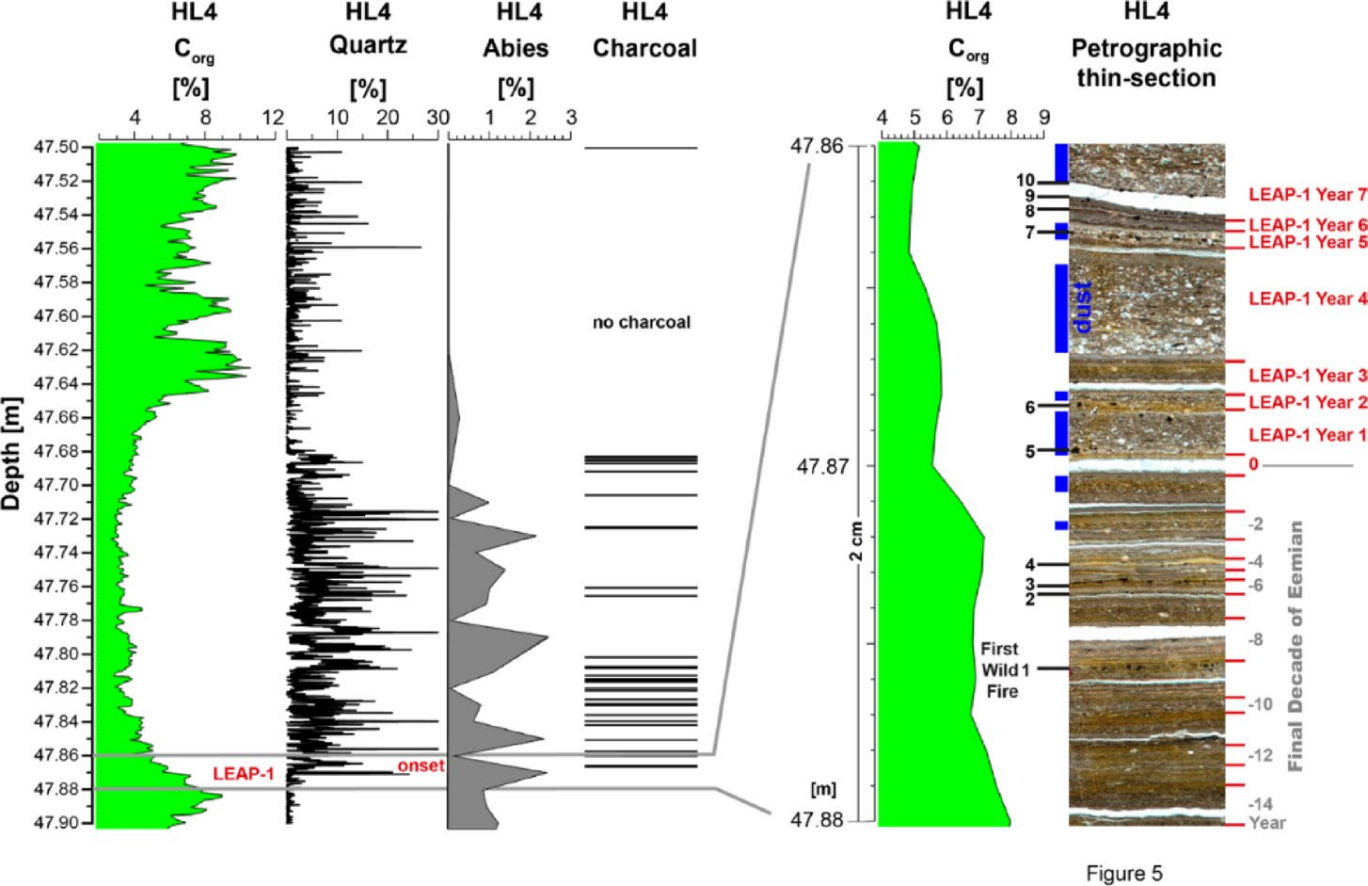
Petrographic thin section and associated time series spanning the final decade of the Eemian and the initial phase of LEAP-I. Overview of the LEAP-I interval showing dust layers, *Abies* pollen percentages, and charcoal occurrence. High-resolution view of the transition from the final Eemian decade to the first decade of LEAP-I, illustrating the rapid onset of environmental change.

Quartz grain-size distributions were determined using the RADIUS method, which analyses petrographic thin sections at 200-µm intervals and detects particles between 20 and 200 μm in diameter^38^. The resulting grain-size records are presented in Fig. 4, with particular focus on the onset of LEAP-I in Figs. 4 and 5. Particles are identified as coloured grain surfaces in polarized-light images, allowing mineralogical classification as well as calculation of size and shape parameters. Based on these measurements, standard grain-size statistics, including sorting, skewness, kurtosis, and mean grain size, are calculated. In addition, particle elongation and surface roughness are determined for each grain. The current version of the RADIUS method also enables the simultaneous detection of dark organic particles and charcoal fragments.

### Charcoal

Charcoal was identified using three independent approaches. Charcoal particles typically display sharp, angular boundaries, although some grains are partially coated by small pyrite framboids (Supplementary Fig. 9).

1. Charcoal layers were identified visually in petrographic thin sections and incorporated into the varve-counting record.
2. The same layers were independently detected by the RADIUS analysis of the petrographic thin sections.
3. To verify charcoal identification, charcoal particles were additionally counted on pollen slides, where pyrite is removed during sample preparation.

All three approaches produced highly consistent results. Figure 3 presents the charcoal record derived from varve counting, whereas Fig. 4 shows the RADIUS-based results. An example of a charcoal particle without pyrite coating, photographed on a pollen slide, is shown in Supplementary Fig. 9.

## Results

### Climate and environment during the Late Eemian

The Eemian sediments in all cores are disrupted by the large Slump A, which occurs within the late Eemian *Carpinus–Abies* pollen zone. The homogenized slump deposit is most clearly visible in core EI2 between 47 and 49 m depth (Supplementary Fig. 2). Although the slumping event occurred within the *Carpinus–Abies* zone, it was sufficiently extensive to incorporate sediments from the underlying pollen zone (Fig. 2).

Varve counting indicates that Slump B occurred approximately 600 years before the onset of LEAP-I. At both Hoher List and Jungferweiher, this major slumping event is associated with volcanic ash deposits. The slump may therefore have been triggered by earthquakes related to volcanic activity, although a substantial lake-level decline cannot be excluded. It is noteworthy that this event coincides with the initial transition towards cooler conditions recorded in Greenland ice-core records^21^(Fig. 3), although the chronological uncertainties do not allow a direct correlation.

### Climatic and environmental evolution at the end of the last interglacial

The key phases of the Last Glacial Inception are preserved between 48 and 45 m depth in core HL4 and span at least 2700 varve years within the floating chronology (Fig. 3). Because the amount of sediment potentially removed during slumping events cannot be quantified, the total duration of the transition may have been longer. The C_org_ (*chlorins*) record is characterized by high values during the late Eemian *Abies* expansion, followed by two intervals of markedly reduced concentrations corresponding to LEAP-I and LEAP-II. Both LEAP phases exhibit similar environmental characteristics, including reduced tree abundance, expansion of grassland, charcoal layers, and aeolian dust deposits, all indicating increased aridity. Summer calcite precipitation continued throughout both intervals, suggesting that summer temperatures remained relatively high.

LEAP-I lasted approximately 250 years and culminated in the disappearance of *Abies* from the regional vegetation (Fig. 3). Forest vegetation subsequently recovered and persisted for approximately 750 years until the onset of LEAP-II. This recovery phase remained broadly similar to the preceding Eemian forest, although *Abies* did not re-establish. Varve structure during this interval was comparable to that observed before LEAP-I, with abundant diatoms and annual summer calcite precipitation (Supplementary Fig. 10).

LEAP-II was characterized by the same indicators of aridity observed during LEAP-I but persisted for approximately 750 years. Following LEAP-II, vegetation recovered only partially, developing into a boreal forest dominated by *Pinus*, *Betula*, and *Picea* that lasted for approximately 350 years. Summer calcite precipitation continued during this phase, although calcite layers were generally thinner than during the preceding LEAP intervals. The boreal forest phase terminated with Slump D, which consists of varved but strongly microfolded sediments. These deposits may represent reworked material derived from the LEAP-II interval. Because the folded sediments cannot be counted reliably, this section is excluded from the varve chronology, and the potential loss of annual layers cannot be assessed.

LEAP-III, the final environmental transition, was accompanied by a major lake-level decline at Hoher List. The associated slump deposit contains large charcoal fragments, indicating wildfire activity in the immediate surroundings of the maar lake. The duration of LEAP-III cannot be determined because erosion may have removed part of the sedimentary record. Nevertheless, this interval marks a pronounced transition towards substantially more arid and cooler environmental conditions, as indicated by the complete absence of summer calcite precipitation during the centuries following LEAP-III.

Table 2. Summary of the succession of environmental phases identified during the Last Glacial Inception.

**Table 2:** Succession of environmental phases during the LGI.

Phases	Environmental change during the Last Glacial Inception (LGI)	Years
	Frequent dust layers, no charcoal, quite likely C25	
12	Landscape with abundant grass and scattered pine and birch	
11	Slump D with reworking of older LEAP-II sediments and large charcoal	?
10	Spead of *Pinus* and *Picea*, boreal forest	350
9	Slump C, most likely erosional with loss of annual layers	?
8	Onset of LEAP-II	750
7	Forest with *Quercus*, *Picea* and *Carpinus*, warm summer	750
6	End of LEAP-I after 250 years duration	
5	Disappearance of *Abies*, 200 years after the onset of LEAP-I	
4	Onset of LEAP-I with frequent wildfire and dust deflation	250
3	End of Slump B, at minimum 600 years before onset of LEAP-I	600
2	Volcanic Ash (eruption in the Eifel volcanic field)	
1	Slump A in the later *Carpinus*-*Abies* Phase	
	Eemian interglacial forest for about 10,000 years	
	**Duration of all phases from Slump A to Slump D**: (more than)	2700

Figure 4 documents the grain-size characteristics of the LEAP-I interval at 200-µm resolution using the RADIUS method. Prior to LEAP-I, varve thicknesses are generally below 1 mm yr⁻¹, whereas values increase to several millimetres during LEAP-I. Organic particles visible in thin sections account for less than 5% of all detections, while charcoal is restricted almost exclusively to the LEAP interval. Maximum quartz grain sizes consistently exceed 50 μm during LEAP-I and occasionally reach 150 μm. Sorting and roundness values remain high throughout the interval. This combination of grain-size characteristics is typical of locally deflated cover sands. The particles are considerably coarser than typical loess deposits, which are generally silt-sized and transported over longer distances. The fine-sand fraction deposited in the Hoher List maar lake most likely reflects local aeolian deflation and short-distance transport by near-surface winds exceeding approximately 6 m s⁻¹, corresponding to the modern threshold for dust deflation. This interpretation is consistent with observations from other ELSA sediment records^39^.

The interval between approximately 120,000 and 117,000 BP can therefore be subdivided into ten environmental phases (Table 2), documenting a transition of at least 2700 years in duration, excluding any time potentially lost during slumping events. During this interval, the late Eemian landscape evolved from a temperate mixed forest to a cold grass-dominated steppe with scattered trees.

All LEAPs are characterized by a marked decline in *Abies* pollen percentages and coincides with the first occurrence of charcoal layers. These charcoal particles are small, well sorted, and concentrated within discrete layers, indicating regional wildfire activity across the Eifel. Modern silver fir (*Abies alba*) is highly sensitive to fire^40^, and wildfire occurrence is commonly associated with drought conditions. The onset of LEAP-I therefore likely reflects a significant increase in aridity. Additional evidence for increasing aridity is provided by layers containing extremely well-sorted clastic particles (Figs. 4 and 5). Grain-size analyses of core HL2 previously identified these particles as aeolian dust deposits^14,41^.

## Discussion

### Comparison of the eemian records from the Eifel with other Central European climate archives

Classical Eemian pollen records, including those of Zagwijn^34^, Woillard^35^, Müller^32^, Menke^33^, and Litt et al.^36^, consistently document a final expansion of *Abies* accompanied by abundant thermophilous tree taxa, followed by assemblages dominated by *Pinus*, *Betula*, and Poaceae. However, the timing of these transitional phases has generally remained poorly constrained in the older records.

A recent re-evaluation of the Bispingen profile^42^ reached similar conclusions and estimated the duration of the Eemian forest phases, including their terminal stage, to be approximately 13,000 years. This estimate is consistent with the chronology proposed here. In the Bispingen record, however, the final termination of the interglacial is preserved only within sandy deposits, which may represent either aeolian cover sands or reworked dune sediments.

The aeolian deposits at both Bispingen and Hoher List were likely formed under strong near-surface winds that promoted saltation, as reflected by the high roundness values of the transported grains (Fig. 4). These deposits therefore differ fundamentally from typical loess, which is transported in suspension and can be carried over considerably greater distances. Loess deposits are widespread across central Europe, and several have been dated by luminescence methods to the millennium associated with North Atlantic cold event C25^43,44^, in good agreement with the HL4 chronology.

Pollen profiles from Poland also reveal comparable succession, but with slight differences in the definition of local pollen zones^45,46^, however indicators of a local humid phase and two cooling phases during the terminal Eemian, quite comparable to LEAP-I, II, III. Other evidence for the end of the Eemian comes from a marine core from the coastal region of northern Denmark. Evidence for a synchroneity with North Atlantic hydrographic changes is obvious from the marine fauna of these cores, and strikingly, the marine records also reveal three pulses, marking the transition from MIS5e to MIS5d, called Event S1-S3 by the authors^47^.

### Timing the onset of LEAP-I

Despite the unprecedented detail preserved in the HL4 varved sediments, a precise absolute age for the onset of LEAP-I remains unattainable due to the absence of directly applicable absolute dating methods for lake sediments. We have however two stratigraphic informations, which bracket the LGI. The pollen succession in all JW, HL, EI cores reveal the typical Eemian taxa of a mixed forest, however disturbed by the Slump-A and -B before the final *Abies* expansion.

The relation of LEAP-I, -II to Han sur Lesse speleothem, as shown in Fig. 3 is based on the observation that the duration from the onset of LEAP-I to the end of LEAP-II is 1750 years according to the varve chronology (Fig. 3a). An arid interval of 1750 years could fit reasonably into the Han sur Lesse δ13C anomaly between 119,550 and 118,100 year b2k, given the relative error of + 700/−1000 years in speleothem datings^7^.

The anchor at LEAP-III places the onset of LEAP-I to approximately 119,100 year b2k on the NGRIP time scale^21^, i.e. at a time when the cooling in the Greenland temperatures and North Atlantic hydroclimate enhanced in an event interrupting gradual temperature decrease, which was ongoing since 121,000 year b2k (Fig. 3b). This long term cooling trend follows the orbitally controlled insolation^21,27^, which places the final LEAP-III at a level comparable to modern summer insolation.

### Rate of climate change during LEAP-I

Of particular importance for understanding the origin of the LEAP events is not only their timing but also the rate at which environmental conditions changed at the onset of LEAP-I. Up to a depth of 47.88 m, varves in core HL4 display a relatively uniform structure characterized by abundant diatoms and regular summer calcite precipitation, reflecting the warm and productive conditions of the late Eemian lake (Supplementary Fig. 10). Comparable varve structures are also observed in Holocene ELSA lake records prior to the onset of intensive human land use.

At 47.86 m depth, the first charcoal layer and the first layer of well-sorted clastic particles appear simultaneously, indicating the onset of wildfire activity and aeolian dust deposition (Fig. 4). During the first decade of LEAP-I, dust layers occur at intervals of approximately two to three years and are commonly associated with evidence of diatom productivity, suggesting deposition during summer conditions. Within only ten years, dust deposition became nearly annual, producing layers several millimetres thick that frequently contain charcoal particles.

Summer temperatures nevertheless remained relatively high, as indicated by the continued occurrence of calcite precipitation (Varve Type 2; Supplementary Fig. 10). The transition from the first evidence of wildfire activity to persistent aeolian dust deposition therefore occurred within approximately two decades and was characterized primarily by increasing aridity rather than by substantial summer cooling.

### Possible drivers of aridification

The mechanisms responsible for the three LEAP events must have been capable of producing extremely rapid environmental transitions, shifting the climate system from a stable interglacial state into a distinctly different regime within only a few decades and maintaining these conditions for several centuries.

Persistent drought across central Europe is most plausibly linked to major changes in atmospheric circulation, particularly in the westerly flow originating over the North Atlantic. Most precipitation reaching central Europe ultimately derives from evaporation over the North Atlantic Ocean. Sirocko et al.^15^ previously proposed that abrupt warming events during MIS 3 interstadials were associated with rapid retreats of North Atlantic sea ice.

Changes in sea ice extent are likely to coincide with corresponding weather changes on land. But also the development of snowfields and the initial ice sheet growth on land are processes that could affect the LGI strongly. An experiment with the Hamburg Echam Model indicated indeed that an LGI can be initiated by a perennial snow cover over North America, which could develop at the level of summer insolation reached at 118.000 BP. Once summer melting was no longer sufficient to remove winter snow accumulation, ice-sheet growth became self-sustaining^48^. Although this model simulation reveal a very reasonable process to explain the abruptness of the observed changes in aridity and finally cooling, they do not constrain its precise timing.

LEAP-I may represent an analogous threshold response in northern Europe, potentially involving the persistence of snow cover across Scandinavia during summer. The expected atmospheric response would be the development of a semi-permanent high-pressure system over northern Europe, promoting repeated incursions of cold and dry air into central Europe during spring and summer. Such circulation patterns could have reduced precipitation and enhanced continental aridity while still allowing warm summer surface-water temperatures under high insolation conditions.

The alternation between dust-rich and dust-free summers during the first decade of LEAP-I (Fig. 4) is consistent with such a highly variable atmospheric regime. The final transition towards fully stadial conditions did not occur until LEAP-III, when Northern Hemisphere summer insolation had declined further and permanent ice-sheet development commenced in Scandinavia during MIS 5d^49^.

### Vegetation response and ecological sensitivity of *Abies alba*

Many late Eemian pollen records from across Europe document the appearance of *Abies alba* only during the final phase of the interglacial, either as scattered occurrences or as a distinct pollen zone^35,36^. Today, *Abies alba* is widely distributed across Europe and occupies a broad range of climatic environments^40^. The species tolerates summer temperatures exceeding 30 °C and occurs in Mediterranean regions, while also enduring winter temperatures below − 25 °C, provided that severe frost does not affect the sensitive early growth period in spring.

In contrast to many other conifer species, *Abies alba* is however particularly vulnerable to fire^40^. Its resin is located directly beneath the bark and can ignite readily, whereas *Picea* and *Pinus* possess resin canals protected within the sapwood. Consequently, *Abies alba* is today uncommon in regions characterized by frequent wildfires. Because wildfire occurrence is commonly associated with drought conditions, the decline and eventual disappearance of *Abies* during LEAP-I may provide an additional indicator of increasing aridity. The coincidence of *Abies* decline, charcoal deposition, and aeolian dust accumulation is consistent with a substantial environmental shift towards drier conditions.

### Potential forcing mechanisms of the LEAPs

A comprehensive synthesis of Last Interglacial climate evolution in the North Atlantic and Europe was recently presented by Tzedakis et al.^2^. Their analysis emphasizes orbital forcing as the primary driver of long-term climate evolution while highlighting the importance of interactions among oceanic, cryospheric, atmospheric, and terrestrial systems in shaping regional responses.

The mechanisms responsible for changes in North Atlantic circulation, and their specific impacts on the volcanic landscapes of the Eifel region, remain incompletely understood. Many available paleoclimate archives lack the temporal resolution required to resolve decadal-scale processes, while others remain difficult to date with sufficient precision.

Future high-resolution ice-core reconstructions of greenhouse-gas concentrations, atmospheric dust fluxes, cosmogenic radionuclides such as ¹⁰Be, and geomagnetic-field variability may provide important constraints on the forcing mechanisms involved. The HL4 record itself does not directly identify the ultimate cause of the LEAPs but establishes important boundary conditions by demonstrating that the Last Glacial Inception lasted at least 2700 years and included two prolonged intervals of pronounced aridification lasting approximately 250 and at least 750 years, respectively.

However, only a limited number of these processes are capable of producing environmental changes on the timescales documented by the HL4 record. In particular, changes in North Atlantic hydrography provide a plausible mechanism for explaining the abrupt onset of the LEAPs. Independent evidence indicates a weakening of the Atlantic Meridional Overturning Circulation (AMOC) towards the end of the Last Interglacial^50^, making this process a potential contributor to the observed environmental instability.

## Supplementary Information

Below is the link to the electronic supplementary material.

Supplementary Material 1

Supplementary Material 2

## Data Availability

The data is available on the ELSA website (elsa-project.de) and PANGAEA data repository.
