# Supplementary material for: Annual-resolution evidence for aridity spikes at the onset of the Last Glacial Inception in an Eifel maar lake sediment
Source: Sci Rep. 2026 Aug 15;16:25526. doi: 10.1038/s41598-026-64586-6 (PMC13477475; doi:10.1038/s41598-026-64586-6)
Supplement: Supplementary file 1 [file 41598_2026_64586_MOESM1_ESM.pdf]

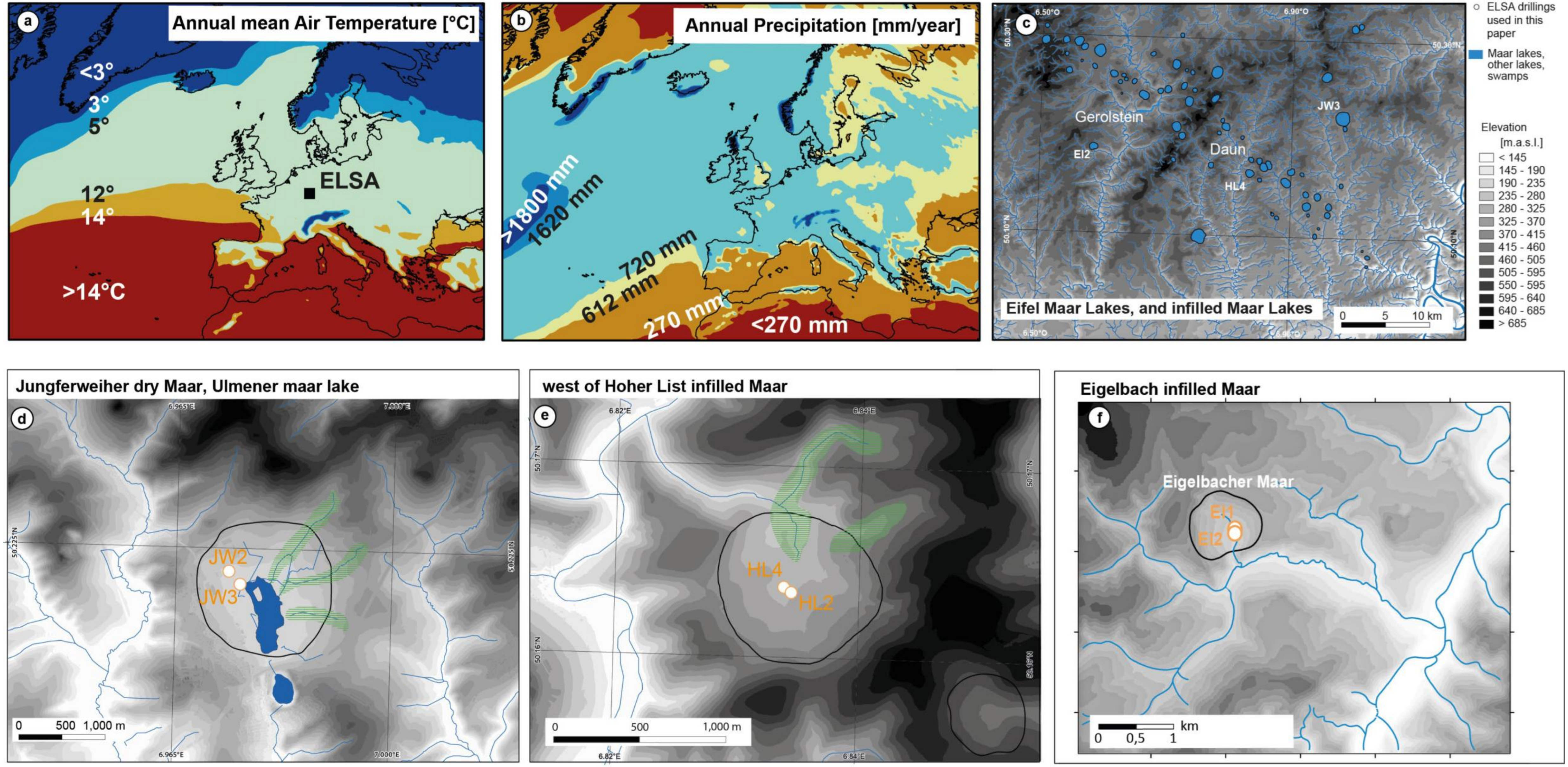

Supplementary Figure 1. a) Modern annual mean temperature of the north Atlantic and Europe for the time 1980–2010. **b)** Respective data for modern annual precipitation. (**a, b)** Adapted from Sirocko et al. [1]. Reproduced with permission from Springer Nature.) **c)** Digital Elevation Model (DEM) of the Eifel and the distribution of Eifel maar lakes investigated within the last glacial cycle. **d)** DEM of the Jungferweiher maar, with core locations JW2 and JW3. **e)** DEM of the maar west of Hoher List, with core locations HL2 and HL4. **f)** DEM of the Eigelbach infilled maar, including locations of cores EI1 and EI2. (Digital Elevation Models **c–f)** created in QGIS[2] with open-access topography data.)

ELSA core EI2

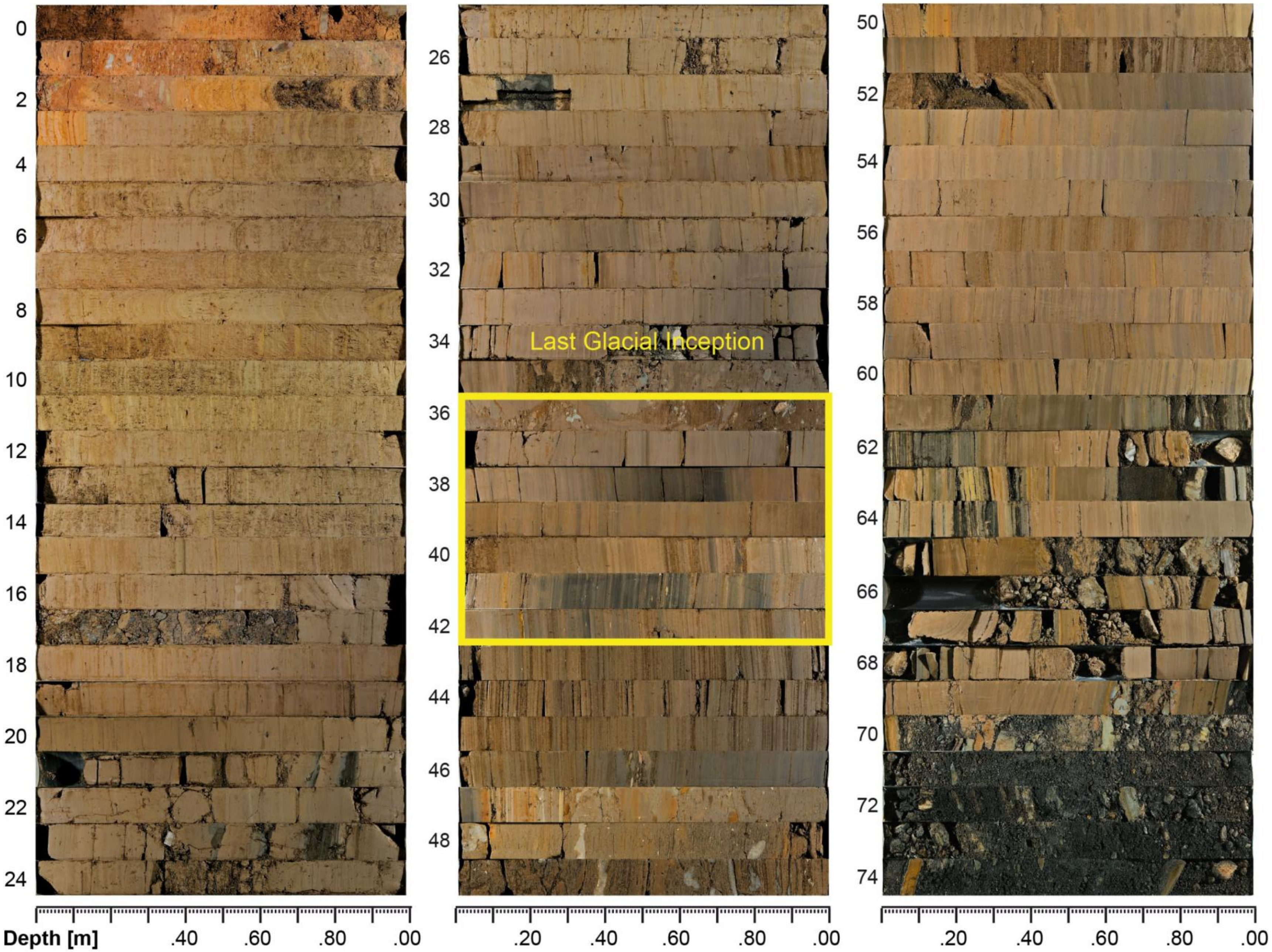

Supplementary Figure 2. Photographic documentation of sediment core EI2 from the Eigelbach infilled maar.

ELSA core JW3

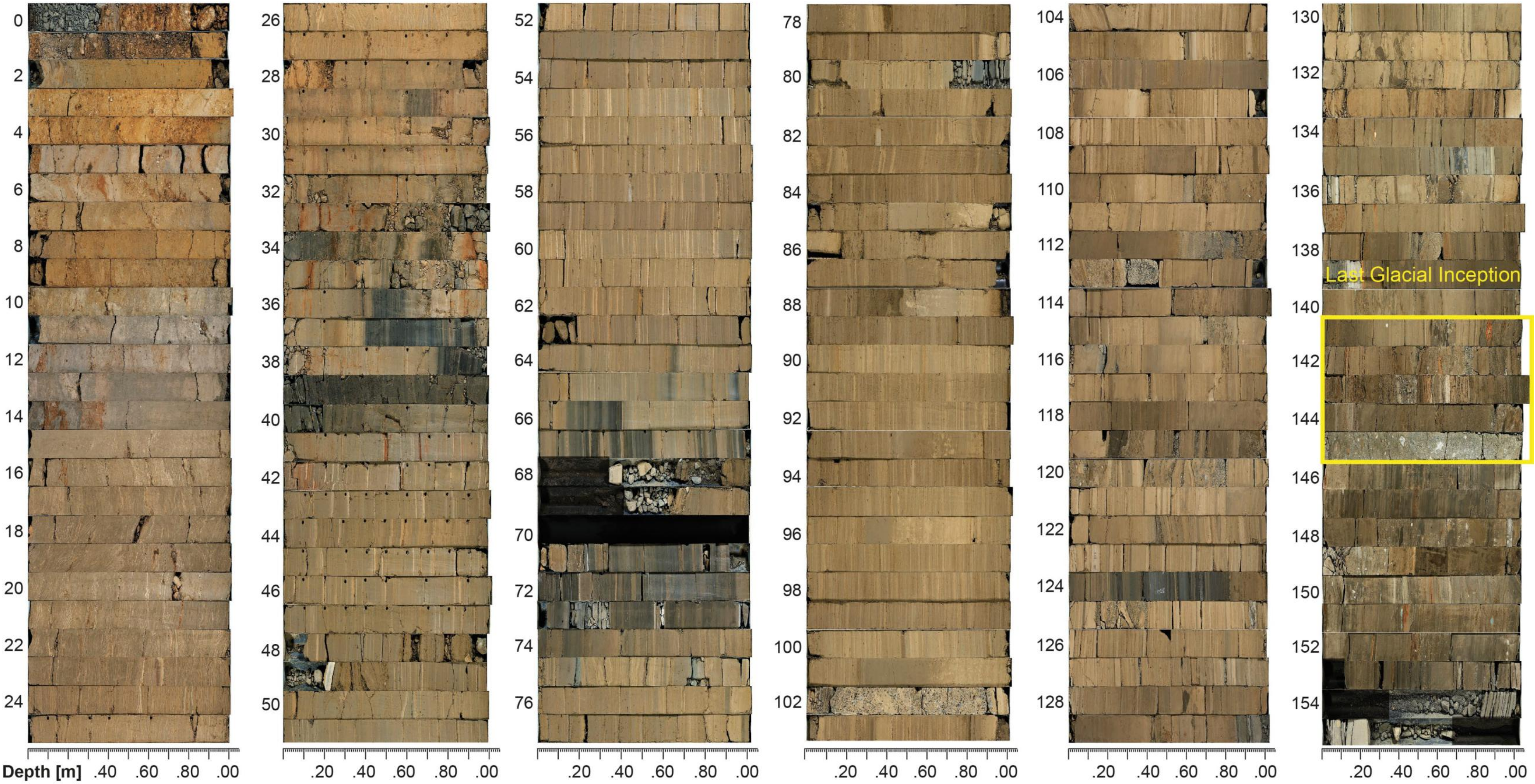

Supplementary Figure 3. Photographic documentation of sediment core JW3 from the infilled maar of Jungferweiher.

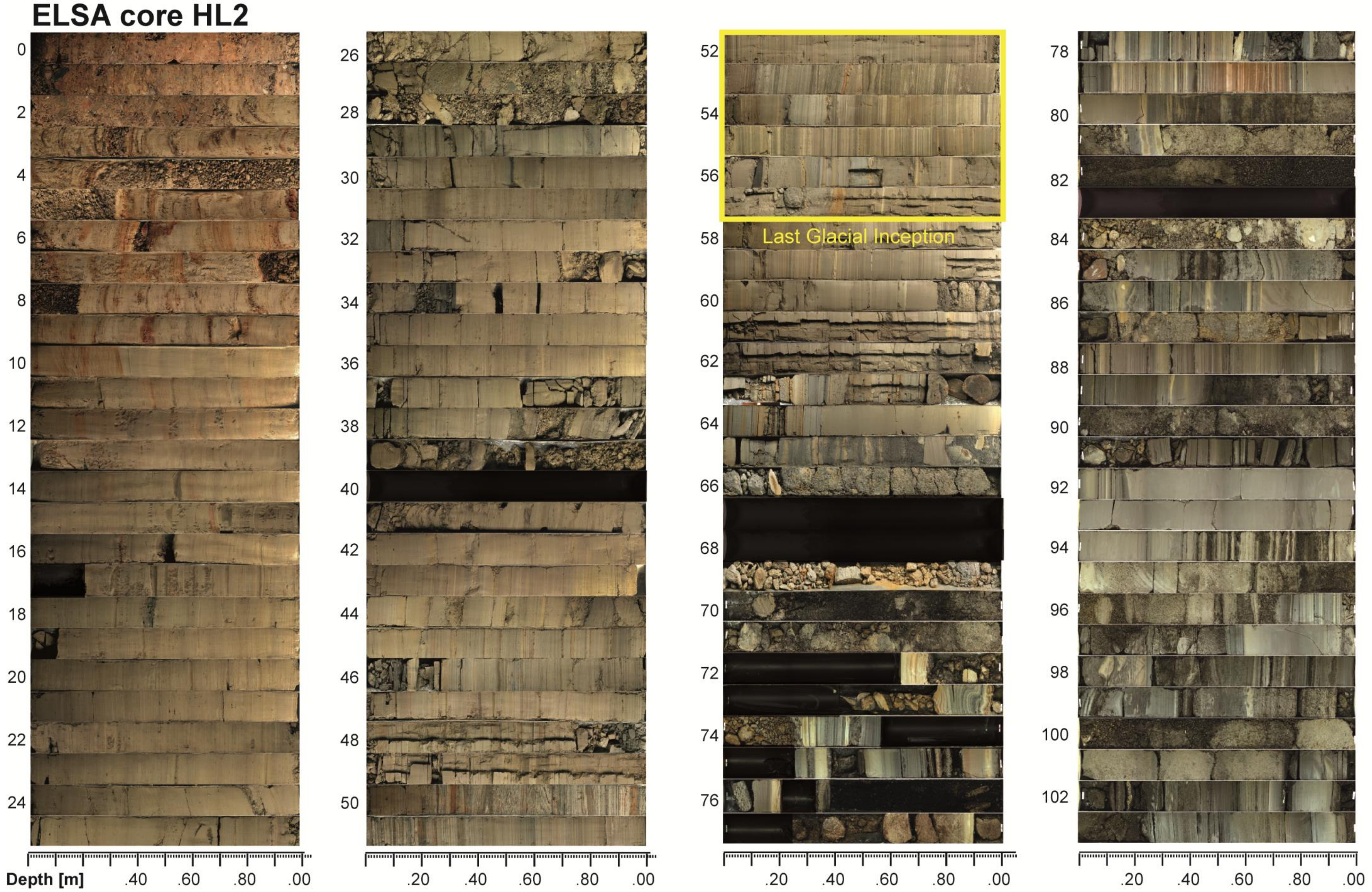

Supplementary Figure 4. Photographic documentation of sediment core HL2 from the infilled maar west of Hoher List.

ELSA core HL4

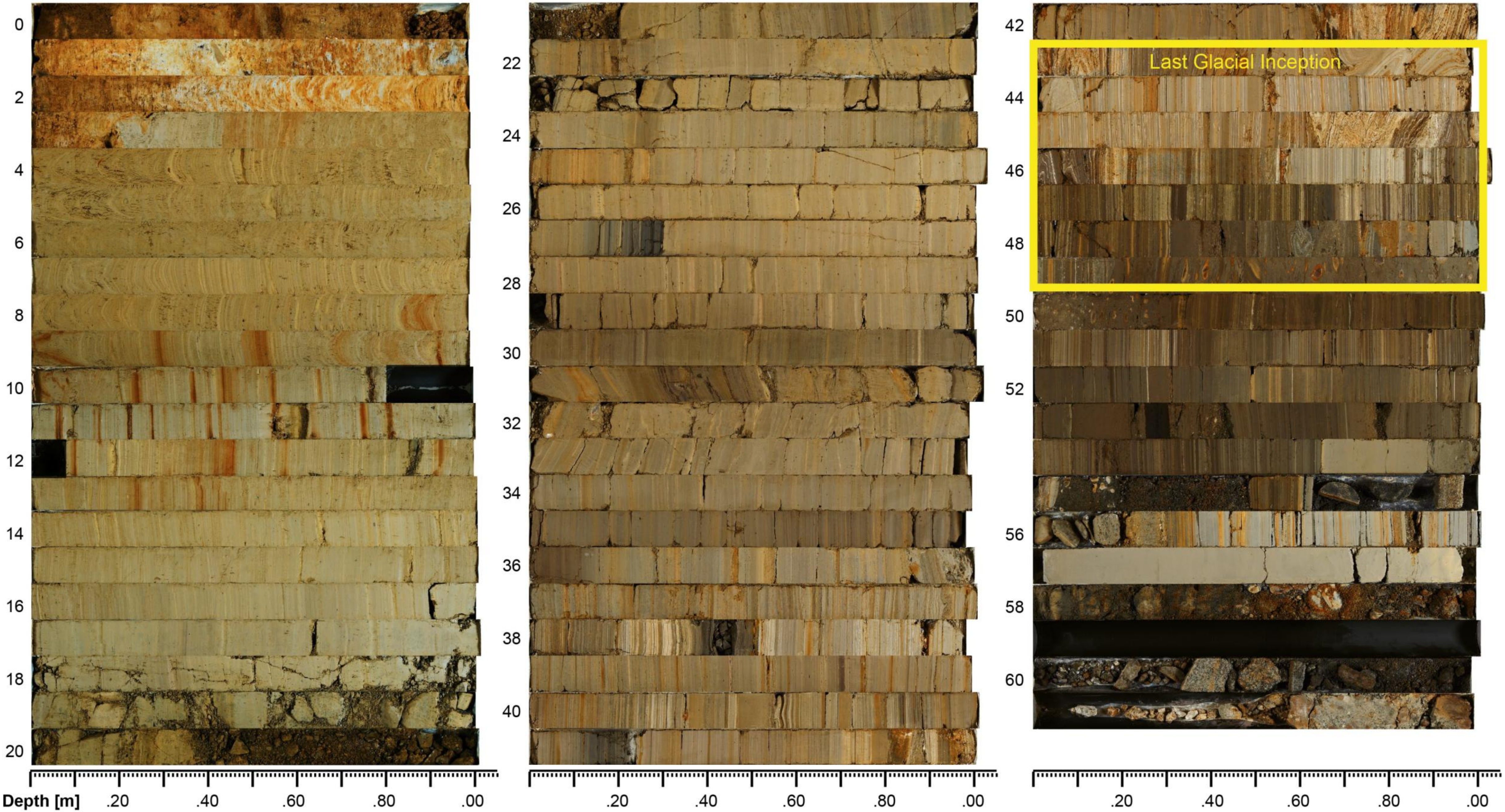

Supplementary Figure 5. Photographic documentation of sediment core HL4 from the infilled maar west of Hoher List.

ELSA core EI2

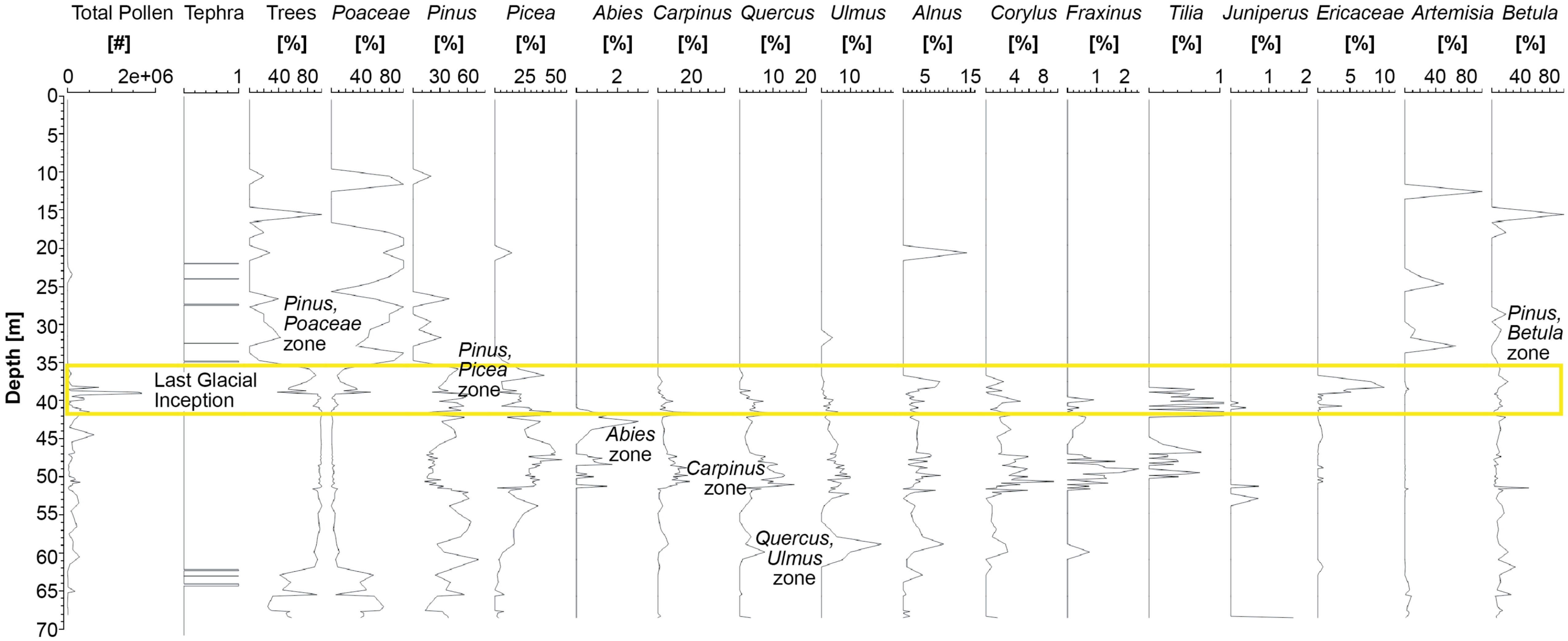

Supplementary Figure 6. Pollen of sediment core EI2 from the Eigelbach infilled maar.

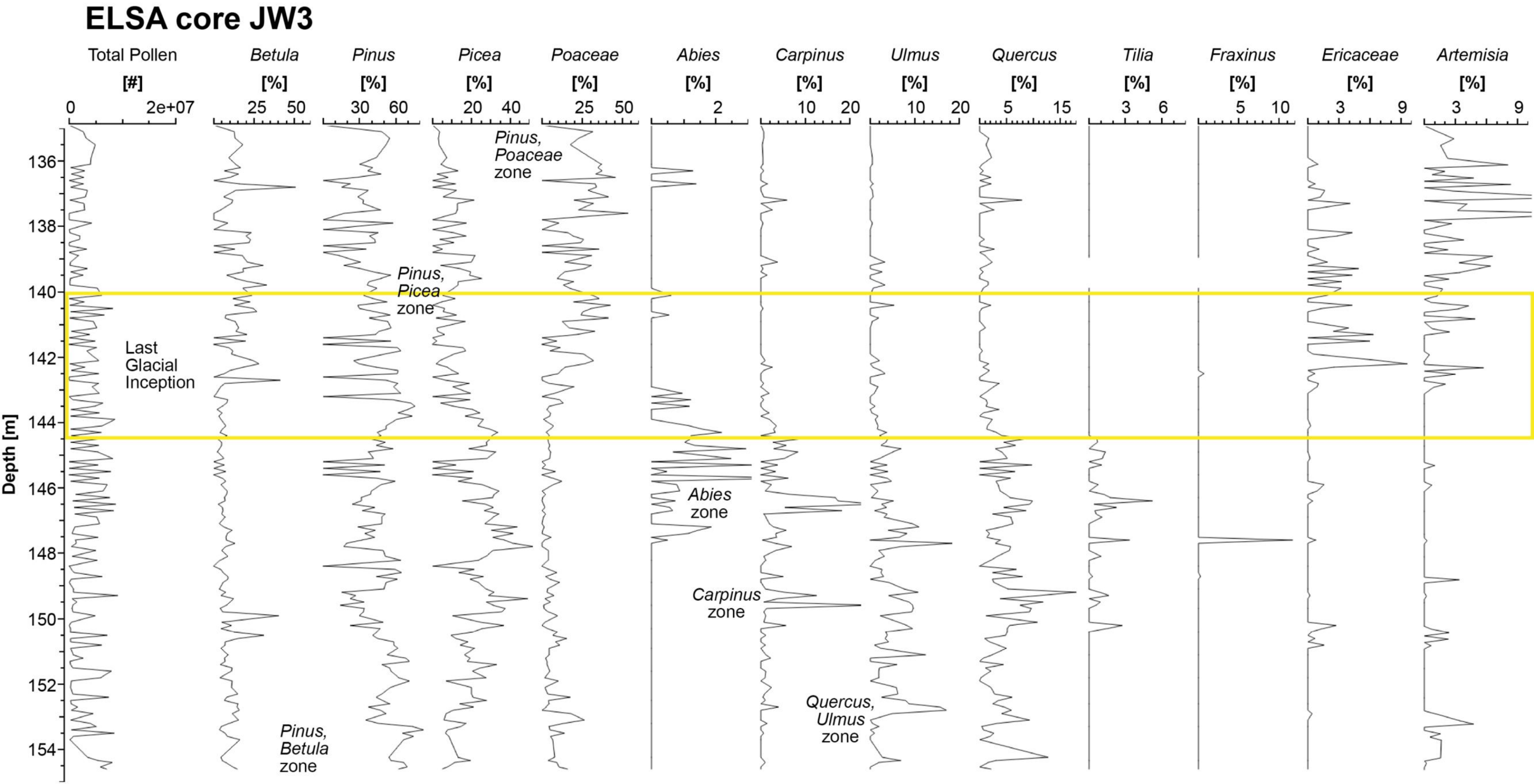

Supplementary Figure 7. Pollen of sediment core JW3 from the infilled maar of Jungferweiher.

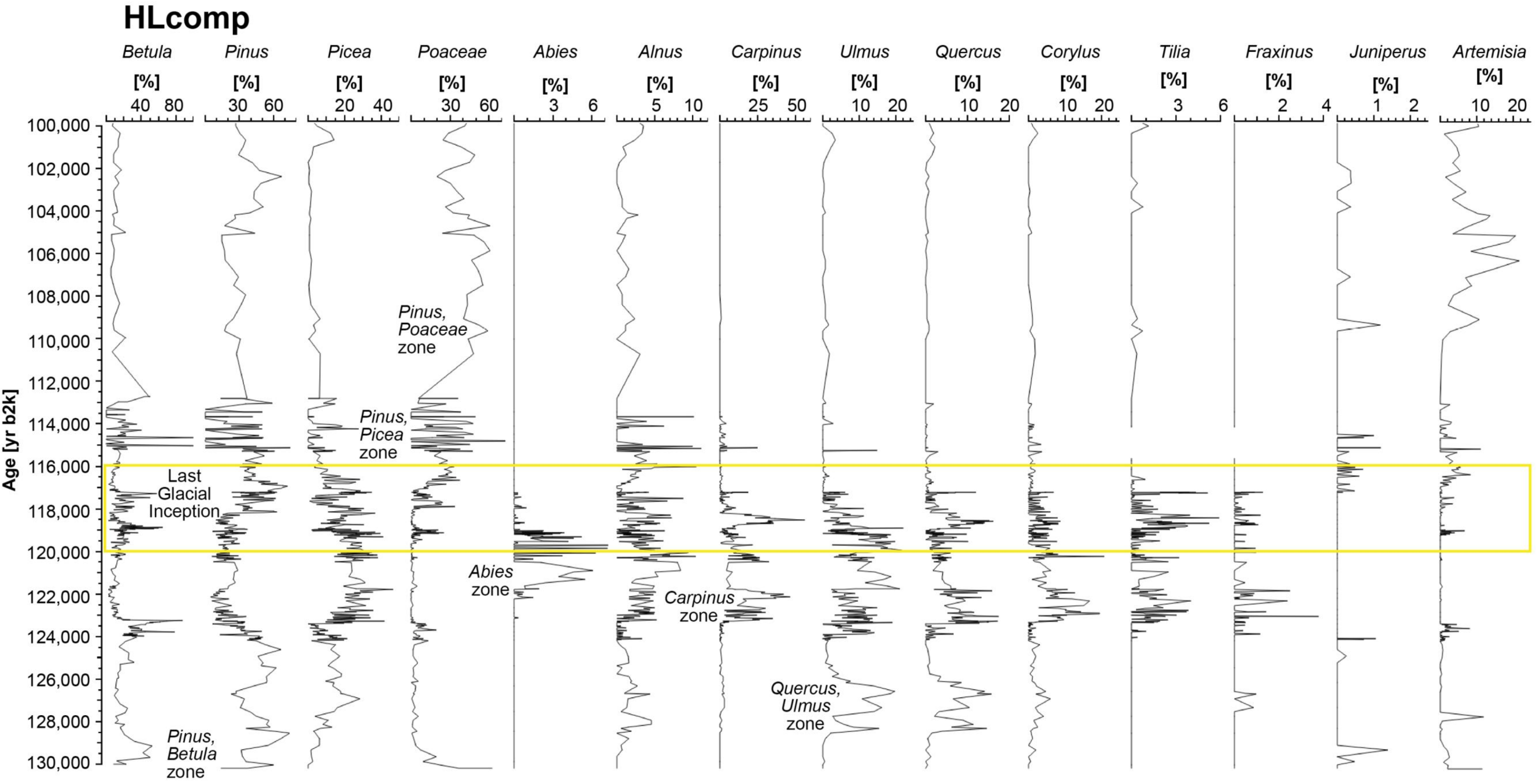

Supplementary Figure 8. Pollen of sediment cores HL2 and HL4 from the infilled maar west of Hoher List.

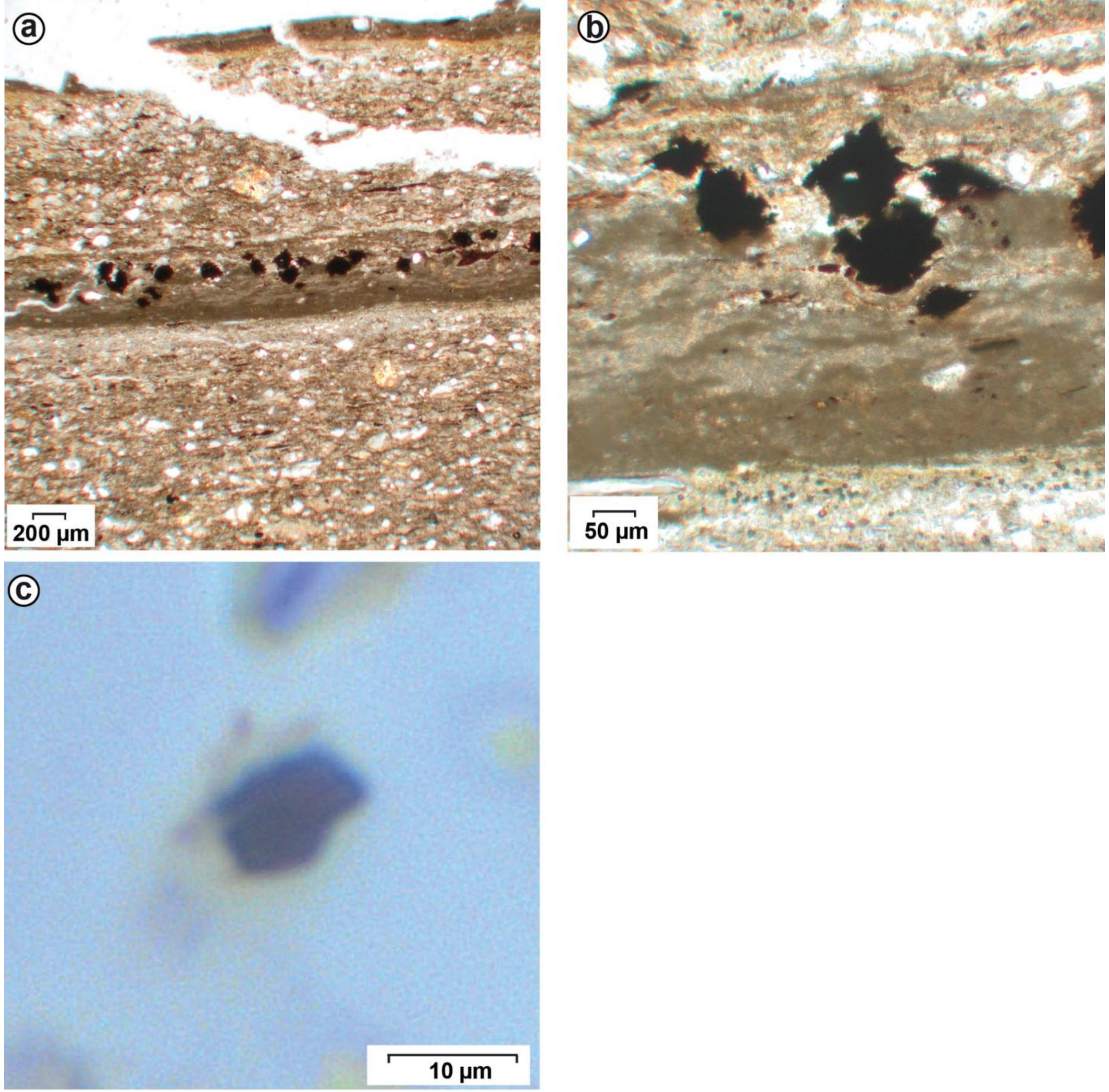

Supplementary Figure 9. Images of charcoal in the petrographic thin section (a, b) and on slides for pollen analysis (c).

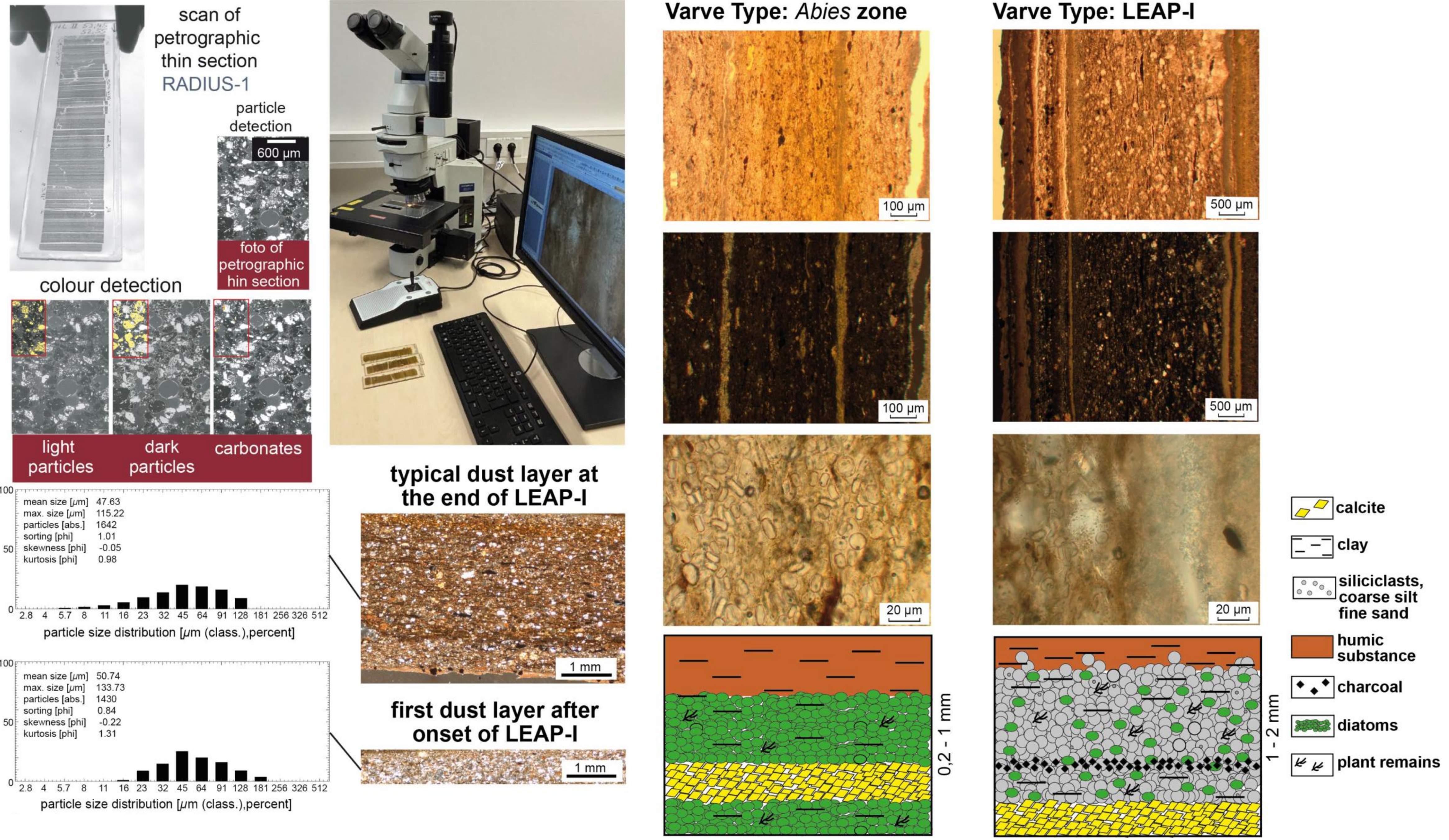

Supplementary Figure 10. Workflow of the RADIUS grain size analysis of petrographic thin sections; Images of dust layers with histogram of the quartz grain size distribution; schematic drawing of the two dominant varve types at the very late Eemian and during LEAP-I.

References

1. Sirocko, F. et al. Muted multidecadal climate variability in central Europe during cold stadial periods. Nat. Geosci. 14, 651–658 (2021).

2. Nyall Dawson et al. qgis/QGIS: 3.34.13. Zenodo (2024). https://doi.org/10.5281/ZENODO.14204331
