# Supplementary material for: Annual-resolution evidence for aridity spikes at the onset of the Last Glacial Inception in an Eifel maar lake sediment
Source: Sci Rep. 2026 Aug 15;16:25526. doi: 10.1038/s41598-026-64586-6 (PMC13477475; doi:10.1038/s41598-026-64586-6)
Supplement: Supplementary file 2 [file 41598_2026_64586_MOESM2_ESM.docx]

**Supplementary Data:**

1. Corg (chlorins) for the HL4 section from 50–43 m
2. Pollen for the HL4 section from 50–43 m
3. Subannual resolution of quartz grain size parameters for the section HL4 47.5–47.9 m versus depth and floating chronology.
